# Variations in Seed and Fruit Traits of the Rare and Endangered Chinese Plant *Lilium tsingtauense* Along Environmental Gradients

**DOI:** 10.1002/ece3.73238

**Published:** 2026-03-09

**Authors:** Wanpei Lu, Anning Ding, Xiao Guo, Pulin Sun, Xinqiang Jiang, Jinming Yang, Hai Wang, Xuebin Song, Qingchao Liu

**Affiliations:** ^1^ College of Landscape Architecture and Forestry, Shandong Key Laboratory for Germplasm Innovation of Saline‐Alkaline Tolerant Grasses and Trees Qingdao Agricultural University Qingdao Shandong China

**Keywords:** environmental factors, fruit development, habitat populations, *Lilium tsingtauense*, seed traits

## Abstract

*Lilium tsingtauense* Gilg is a rare and endangered wild plant, but there is insufficient research on the environmental drivers of intraspecific variation in its seed and fruit traits. To investigate the responses of variations in seed and fruit traits to geographical and soil factors across different habitats, 37 sample plots were selected for investigation and statistics within an elevation range of 200–1000 m in Laoshan, China. Mature fruit and soil samples were brought back to measure soil nutrient content, fruit size, seed number and seed germination rate. The results showed that: (a) There are differences in geographical and soil factors among the habitats of different *L. tsingtauense* populations. There were significant differences in elevation, aspect, light intensity, soil water content, soil electrical conductivity, soil organic matter content and soil total nitrogen content among different populations. (b) Fruiting ability responds more readily to environmental changes than fruit and seed traits do. Fruit length, width and thousand‐grain weight were more stable than number of plump seeds per fruit and germination percentage. (c) Significant positive correlations were observed between longitude, elevation, light intensity, soil water content, soil electrical conductivity and fruit and seed traits, while soil total phosphorus content showed a significant negative correlation with fruit and seed traits. Among these, elevation was identified as a potential key environmental factor driving variations in fruit and seed traits of *L. tsingtauense*. Individuals growing at higher elevations exhibited greater fruit production and higher seed germination rates. These findings reveal the environmental variability in fruit and seed traits of *L. tsingtauense* and its influencing factors, providing important insights for identifying core conservation areas and guiding habitat restoration for this wild resource.

## Graphical abstract

This study is the first study on the response of seed traits to environmental changes in different habitats of *L. tsingtauense*. Different from previous studies focusing on individual growth and biomass accumulation, this study emphasizes the limiting effect of external environmental conditions, namely different geographical factors and soil environment, on the breeding of *L. tsingtauense* by analyzing seed traits.

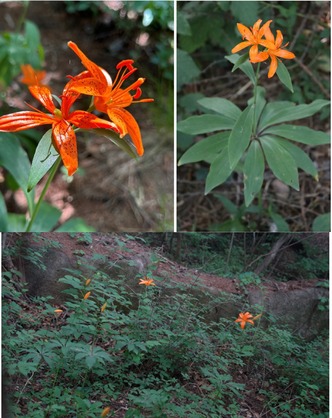

## 1 Introduction

Plant adaptation arises from the interaction between plants and the environment, primarily through two mechanisms: genetic variation or phenotypic plasticity. Compared with genetic adaptation, phenotypic plasticity enables plants to respond more rapidly to environmental changes, thereby allowing them to flexibly cope with such changes (Fox et al. 2019; Zhao et al. 2026). It allows plants to adjust their morphological, chemical, and physiological characteristics under different conditions—such as changes in temperature, light availability, competition, or salt stress—thereby optimizing their survival and reproductive success (Dawson et al. 2024; Ren et al. 2025; Song et al. 2024). Plants are strongly influenced by environmental factors in terms of their growth, resource allocation, and stress response strategies. These variations are reflected in plant traits, and the relationship between plant traits and their environment can be understood directly through trait observations (Famiglietti et al. 2024). Relevant studies have shown that environmental factors significantly influence plant traits (Gong et al. 2023; Liao et al. 2023). For instance, the elementome, as a type of plant trait, can directly exhibit intraspecific variation induced by environmental changes (Guo, Song, et al. 2025; Guo, Sun, et al. 2025); functional traits of subtropical evergreen broad‐leaved forests vary significantly along geographical gradients, with distinct patterns of trait variation across different gradients (Huang et al. 2021); plant traits can shift in response to environmental changes and may display pronounced trait variations across geographical regions (Wang et al. 2020). Trait variation within species is particularly pronounced in response to environmental changes (Guo, Song, et al. 2025; Guo, Sun, et al. 2025). However, most studies have focused on differences in functional traits between species, with only a few delving deeply into the underlying mechanisms of intraspecific trait variation (Kerr et al. 2023; Pritzkow et al. 2020; Urrutia‐Jalabert et al. 2025). Furthermore, many current studies tend to rely on one or a few functional traits to infer plant life strategies (He et al. 2020; Li et al. 2022), overlooking the potentially complex network of relationships among traits (Qiu et al. 2026). Therefore, investigating intraspecific trait variation and the responses of different traits to environmental gradients is crucial for understanding species' adaptive potential and long‐term persistence.

There have been numerous reports on the differences in functional traits and environmental adaptation among Liliaceae plants. For example, salt stress has been shown to affect the growth, morphological characteristics, and physiological traits of flowers and leaves in cut lilies (Kang et al. 2021). Additionally, the heat tolerance of Asiatic lilies has been investigated based on phenological characteristics, stem and leaf morphology, and flowering phenotypes (Bai et al. 2024). These functional traits, serving as indicators of how plants respond to environmental changes (Duan et al. 2022), play a crucial role in the survival and reproductive strategies of Liliaceae species. In studies on the correlation between Liliaceae plants and geographical environmental variations, research has primarily focused on changes in vegetative and floral organs (Macrì et al. 2021). However, studies on the geographical environmental drivers of intraspecific variation in fruit and seed traits of Liliaceae plants have been scarcely reported. Fruit and seed traits play a crucial role in plant reproduction. Fruit traits influence seed dispersal and resource allocation, as well as seedling survival and establishment (Rojas et al. 2022). Furthermore, seed traits can reflect a plant's dispersal capacity, and studies on how seed traits vary across different habitats largely determine a plant's ability to disperse and re‐establish (Si et al. 2022). As the only mobile stage in the plant life cycle, the number of seeds dispersed and the dispersal quality provided to each seed determine the effectiveness of seed dispersal (Tuthill et al. 2023). Traits such as seed size can influence post‐dispersal survival rates (Rosin and Poulsen 2018). Seed germination is a critical stage in the life cycle of flowering plants, influenced by both seed characteristics and external environmental conditions. The germination characteristics of plant seeds and their responses to environmental factors vary among species (Zhang et al. 2020). To promote the growth, reproduction, and population regeneration of Liliaceae plants, further research should investigate the responses of fruit and seed traits to environmental changes. The fruit and seed traits of plants possess significant yet underestimated potential in future ecological research. Investigating the effects of environmental factors on these traits to further explore the mechanisms of plant population regeneration is emerging as a research hotspot in the field of plant ecology (Guo et al. 2024; Paz‐Dyderska and Jagodzinski 2024).

It is estimated that 45% of flowering plant species globally are potentially threatened and may face the risk of extinction (Bachman et al. 2024). To ensure the stabilization and promote the recovery of plant species, an in‐depth understanding of the species' local and national status, distribution dynamics, and the impact of various environmental factors on its populations is essential (Klavina et al. 2025). Furthermore, seed germination traits directly influence the regeneration niche and population persistence, holding significant ecological importance. However, research on the germination characteristics and ecological adaptation strategies of endangered species is extremely limited. This lack of seed‐related information for threatened species hinders the ability to effectively restore ecosystems and protect endangered plants (Ribeiro et al. 2016; Visscher et al. 2022). Therefore, it is both practical and necessary to conduct field community surveys to identify the ecological factors potentially influencing endangered species populations, to predict community response capabilities to environmental changes based on seed traits, and to carry out targeted investigations of seed traits and habitat factors for species with narrow geographic distributions and those classified as threatened on the Red List.

*Lilium tsingtauense* is a rare wild lily with extremely narrow distribution, which is only found in some mountains of Shandong and Anhui provinces in China and some areas of South Korea (Chung et al. 2014). Among them, the core distribution area of *L. tsingtauense* is on the shady slope of Laoshan Mountain at an elevation of 200–1000 m in Shandong Province, China. The current study found that *L. tsingtauense* is now extinct on the island of “Xiaoqingdao,” where its wild resources were first discovered, and its distribution on Laoshan Mountain has also shrunk sharply. Many natural populations of this species have become extinct and have been designated as China's national key protected wild plants (Guo et al. 2011). *Lilium tsingtauense* has important ecological value, ornamental value and economic value. It is a rare leaf group species in the genus Lilium of Liliaceae. The impeller is born in the middle and lower part of the stem, like a rosette, and is an important flower germplasm resource.

In the existing achievements of *L. tsingtauense*, the genes of leaf morphological development (Zhou et al. 2022), chloroplast genes (Lee et al. 2016), and changes of rhizosphere microorganisms with the environment (Liu, Xiao, et al. 2024; Liu, Yang, et al. 2024) were mainly studied. There is still a lack of reports on the response pattern of fruiting characteristics of different habitat populations of *L. tsingtauense* to environmental changes. As a rare and endangered wild plant species, in order to effectively restore and protect the population, it is necessary to have an in‐depth understanding of its reproductive ecological characteristics, and to explore whether the differences in seed traits are related to the environment in which the population is located. Therefore, this study investigated *L. tsingtauense* under different habitats in Laoshan, Qingdao, and measured environmental factors and plant seed traits, aiming to solve the following problems: (a) Is there any difference in the living environment of different populations of *L. tsingtauense*? (b) Is the fruiting ability, fruit and seed traits of *L. tsingtauense* affected by the environment? (c) What are the key ecological factors that lead to differences in seed traits?

## 2 Materials and Methods

### 2.1 Research Area and Sampling Points

#### 2.1.1 Overview of the Study Area

**FIGURE 1:**
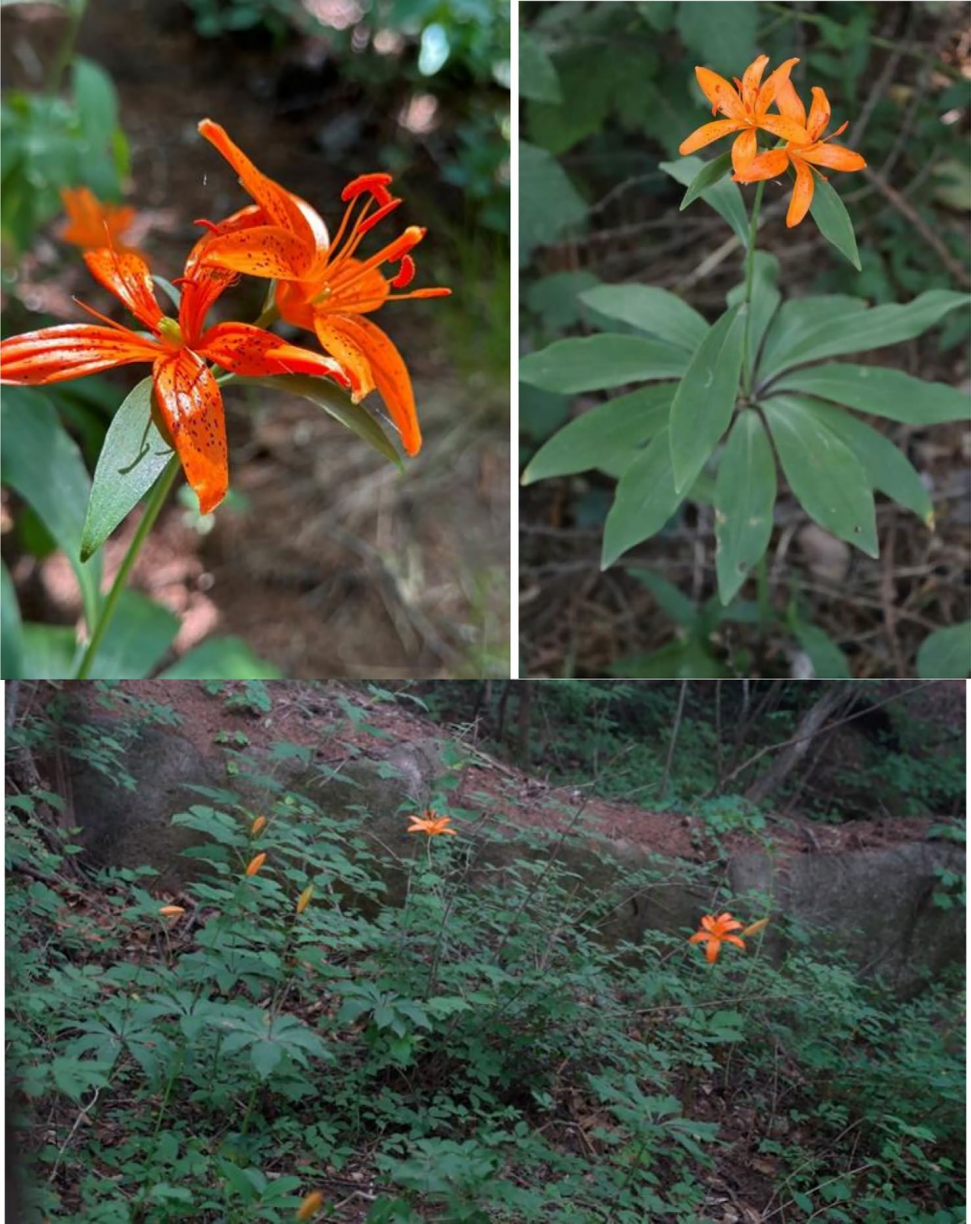
Overall morphology and natural habitat of *Lilium tsingtauense*. *L. tsingtauense* Gilg is a rare species of the section *Martagon* within the genus *Lilium* (Liliaceae). Its leaves are whorled in the middle and lower part of the stem, forming a rosette‐like shape. It produces vibrant, star‐shaped flowers. As a nationally protected wild plant in China, its distribution range is extremely narrow. The overall morphology and natural habitat of this species are shown in figure.

Field investigation and sampling were conducted in Laoshan Scenic Area (36°15′–36°21′ N, 120°58′–120°67′ E), Qingdao City, Shandong Province. The average annual temperature in this area is between 11°C and 12°C, the average annual precipitation is about 734.3 mm, the average annual relative humidity is 73%, and the humidity is the highest in July and August. The average annual sunshine hours is 2515.5 h, and the average annual sunshine rate is 57%. Affected by the ocean and terrain, the eastern mountainous area has more precipitation and moist air. The collection of mature fruits and soil samples was conducted in mid‐to‐late October 2023. The meteorological data used in this study were obtained from the Qingdao Municipal Information Network (http://qdsq.qingdao.gov.cn), which recorded an average annual temperature of 14.5°C and an average annual precipitation of 572.9 mm for that year (Figure 1).

#### 2.1.2 Sampling Method

**FIGURE 2:**
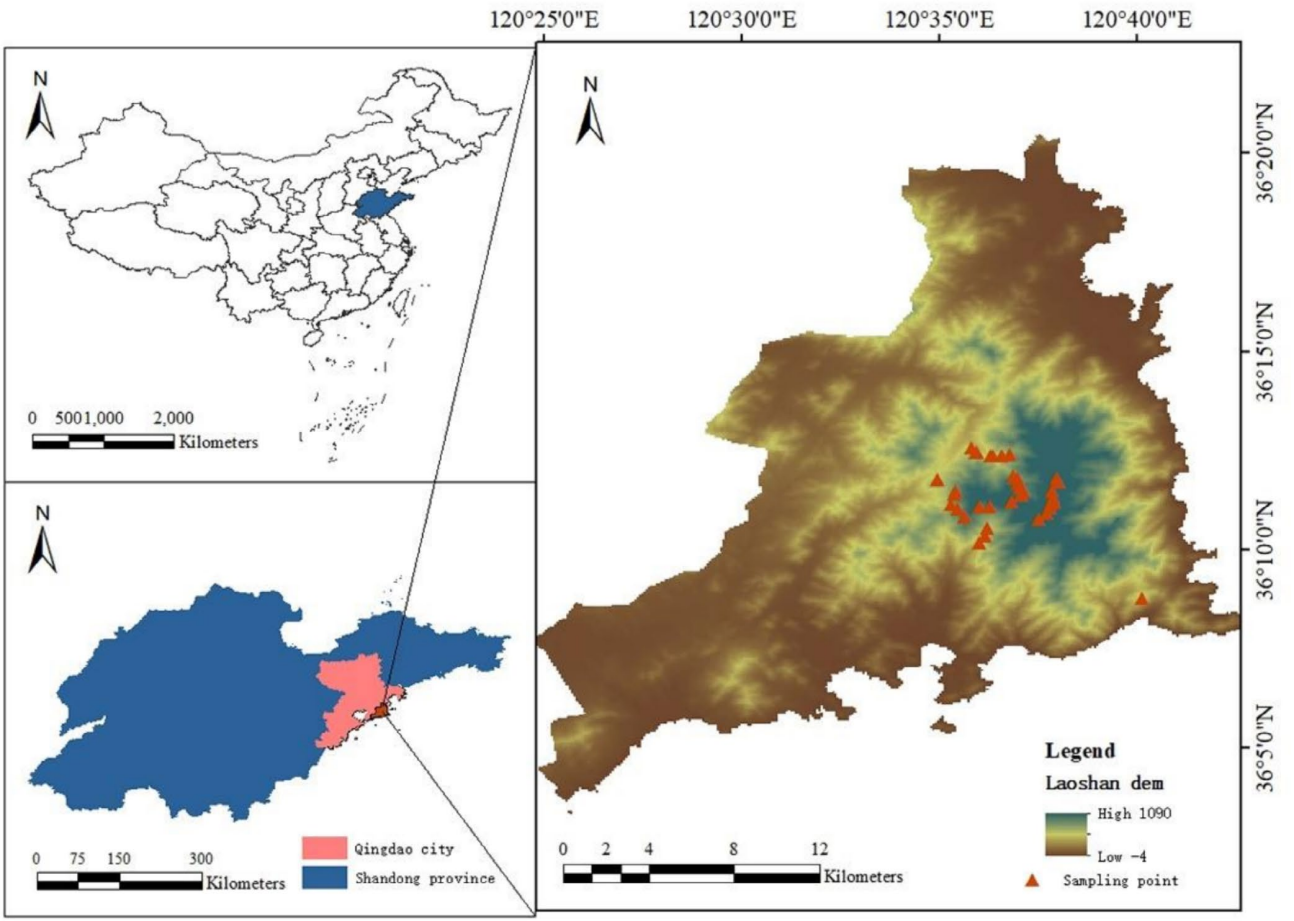
The detailed location of 37 plots on the map.

In mid‐to‐late October 2023, 37 plots were randomly selected for sampling along the elevation of 200–1000 m in Laoshan during the fruit ripening period (Figure 2). The sampling points basically covered the complete distribution area of *L. tsingtauense* in China. At each site, we collected the complete fruit of the plant from a 3 × 3 m quadrat, placed it in a sealed bag and brought it back to the laboratory for natural drying. All soil samples were taken from the growth area of *L. tsingtauense*, close to the plant from the upper soil surface from a depth of 10 cm to about 30 cm, excavated and brought back, sieved by a 2 mm sieve, dried and stored at room temperature for subsequent analysis and determination.

### 2.2 Measurement Method

#### 2.2.1 Geographic Location Acquisition

The Ovi interactive map (Beijing Yuansheng Huawang Software Co. Ltd.) was used to locate the sampling site and obtain longitude, latitude, elevation and aspect data. The light intensity in the growth environment of *L. tsingtauense* population was measured using a SPIC‐300 spectral color illuminometer (Everfine, China). Measurements at each quadrat were taken on clear, cloudless days during periods of daylight between 9:00 and 16:00. Within each quadrat, five growing points of L. tsingtauense were selected, and the probe was placed directly on top of the plants to record the light intensity. The value was recorded once the reading on the illuminometer remained stable for 3 s. The average of these five replicate readings was taken as the mean illuminance for that specific habitat. (Huang et al. 2020).

#### 2.2.2 Determination of Soil Properties

Soil water content (SWC) was measured with freshly collected soil samples, and the soil samples were dried to constant weight at 105°C. The calculation formula is as follows:

(1) $$\mathrm{SWC}=\left(\mathrm{wet}\;\text{soil weight}-\mathrm{dry}\;\text{soil weight}\right)\times 100\%/\mathrm{dry}\;\text{soil weight}$$

The soil samples were air‐dried, ground, and passed through a 2 mm sieve to obtain fine air‐dried soil to measure soil pH and electrical conductivity (EC). Put 0.3 g of dry soil into a 50 mL centrifuge tube, add the corresponding proportion of distilled water to mix, stir, and mix with a magnetic stirrer, and stand for 30 min. The pH value of the upper suspension was determined by a pH meter (PHS‐3C type, Lei Ci, Shanghai, China). After the pH was measured, distilled water was added to the centrifuge tube and mixed well with the same method. The conductivity of the suspension was measured by a conductivity meter (DDSJ‐308A, Lei Ci, Shanghai, China). The soil‐water ratios for measuring pH and conductivity were 1:2.5 and 1:5, respectively (Wu et al. 2025).

Soil organic matter (SOM) was determined by potassium dichromate oxidation method after digestion with sulfuric acid‐nitric acid by graphite digestion instrument (SH420F, Manon, Shandong Haineng Scientific Instrument Co. Ltd.) (Hou et al. 2023; Liu et al. 2025). Total nitrogen (TN) was determined by automatic Kjeldahl nitrogen analyzer (K9860, Manon, Shandong Haineng Scientific Instrument Co. Ltd.) and total phosphorus (TP) was determined by molybdenum‐antimony‐scandium colorimetry (Yuan et al. 2022).

#### 2.2.3 Measurement of Fruit‐Related Indicators

The fruits collected in each quadrat were counted, the number of fruits (Nf) was recorded, the fruit traits were measured, the fruit size was divided, and the large fruit rate (Lr), medium fruit rate (Mr), and small fruit rate (Sr) were calculated. Fruit traits included fruit length (Fl), fruit diameter (Fw), fruit shape index (Fsi), number of plump seeds per fruit (Nsp), number of unfilled seeds per fruit (Nsu), and seed number per fruit (Ns).

The fruit length (Fl) and fruit diameter (Fw) were measured by vernier caliper (Eriezer Tools Co. Ltd., Germany, measurement range: 0–150 mm, measurement accuracy: 0.01 mm). Fruit length (Fl) is the maximum length of the longitudinal section of fresh fruit; fruit diameter (Fw) is the maximum length of the transverse section of fresh fruit; to calculate the fruit shape index (Fsi), the calculation formula is:

(2) Fsi=Fl/Fw

Among them, the fruit transverse diameter > 25 mm is classified as large fruit, the fruit transverse diameter between 20 and 25 mm is classified as medium fruit, and the fruit transverse diameter < 20 mm is classified as small fruit. The ratio of the number of large, medium, and small fruits to the number of fruits in the plot (Nf) is the rate of large, medium, and small fruits.

The collected fruits from each quadrat were placed in the laboratory. After they had naturally dried and cracked, the number of plump seeds, the number of unfilled seeds, and the total number of seeds were recorded individually for each fruit collected from the quadrat. The average values were calculated as the number of plump seeds per fruit (Nsp), the number of unfilled seeds per fruit (Nsu), and the number of seeds per fruit (Ns) for that quadrat.

#### 2.2.4 Measurement of Seed‐Related Indicators

One hundred full seeds of each sample were weighed on the balance with an accuracy of 0.001 g and repeated 8 times. The mean and standard deviation of 8 replicates were calculated and converted to 1000 seed weights to calculate the thousand‐grain weight (TGW) (Fang et al. 2025).

The plump seeds collected from each quadrat were mixed thoroughly and randomly sampled for the seed germination experiment. Three replicates were set up for each quadrat, with 50 healthy seeds randomly selected for each replicate. Without any pretreatment, the seeds were placed in covered glass Petri dishes (120 mm in diameter) lined with a layer of moistened filter paper. (Liu et al. 2023). Referring to the study by Zhang Lingyun (Zhang 2014) on the seed germination characteristics of *L. tsingtauense*, the seeds were cultured in a light incubator (MGC‐250P; Shanghai Yiheng Scientific Instrument Co. Ltd., Shanghai, China) at a temperature of 20°C under dark conditions with a relative humidity of 80% to ensure consistent germination conditions. Radicle protrusion of 1–2 mm from the seed coat was used as the criterion for germination. Seed germination was observed and recorded daily, with appropriate water supplementation provided. The experiment was terminated after no further seed germination occurred for three consecutive days. The calculation formula of seed germination percentage (GP) was as follows:

(3) GP=number of germinated seeds/number of test seeds×100%

### 2.3 Data Analysis

Data were recorded and processed using Microsoft Office Excel 2019. The sampling location map was created using ArcGIS 10.8.1 (Esri, Redlands, California, USA). One‐way analysis of variance (ANOVA) was performed using SPSS 26.0 (SPSS Inc., Chicago, Illinois, USA) to determine significant differences, with the significance level set at *p* < 0.05. Data were assessed prior to ANOVA to ensure they met the assumptions of independence and normality of residuals, and response variables were transformed when necessary. Statistical analyses were conducted using RStudio (Posit Software, PBC, Boston, Massachusetts, USA). To control the false positive rate arising from multiple comparisons, the Benjamini‐Hochberg (BH) method was applied to correct the raw *p*‐values from all relevant tests for false discovery rate (FDR), yielding adjusted *p*‐values (i.e., *q*‐values). Significance was defined as FDR *q* < 0.05. Based on the corrected significance results, Pearson correlation coefficients were calculated using Origin 2022 (OriginLab Corporation, Northampton, Massachusetts, USA) to correlate various environmental variables influencing the seed and fruit traits of *L. tsingtauense* (Ali et al. 2023), and a correlation heatmap was generated.

Redundancy analysis (RDA) was performed using Canoco 5 software (Microcomputer Power, Ithaca, New York, USA). Stepwise linear regression analysis was employed to further explore the main factors contributing to differences in individual seed and fruit trait indicators. Data analysis for constructing the linear regression model was performed using SPSS 26.0 (SPSS Inc., Chicago, Illinois, USA); a larger *R* ^2^ indicated a good fit of the model equation. The stepwise linear regression charts were created using GraphPad Prism 10 (San Diego, California, USA).

## 3 Results

### 3.1 Relationships Between Environmental Factors and Seed and Fruit Traits

The size of the coefficient of variation reflects the size of the variation range. The higher the coefficient of variation, the greater the variability within the group. In this study, the coefficient of variation of longitude and latitude was the lowest, 0.01% and 0.03%, respectively, in the statistics of the characteristics of environmental factors of *L. tsingtauense* (Table 1), that is, there was almost no difference between longitude and latitude. Except for the coefficient of variation of soil pH was 6.23%, and the coefficient of variation of soil total phosphorus content, which was 10.99%, the coefficient of variation of other environmental factors was greater than 30%, indicating that the variation of soil pH and soil total phosphorus content was small, and the two factors were relatively stable.

**TABLE 1:** Statistics on the characteristics of environmental factors of *L. tsingtauense*.

Index	Maximum	Minimum	Mean	SD	CV (%)
Geographic factors					
Longitude	120.67	120.58	120.61	0.02	0.01
Latitude	36.21	36.15	36.17	0.01	0.03
Aspect	360	1	195.54	116	59.32
Elevation (m)	989.65	334.39	661.34	223.62	33.81
Light intensity (lx)	7774.6	1500	4037.09	1527.14	37.83
Soil factors					
Soil pH	5.82	4.67	5.14	0.32	6.23
Soil water content (%)	39.38	3.51	20.23	9.32	46.06
Soil electrical conductivity (ms/cm)	0.77	0.05	0.2	0.18	90.93
Soil organic matter content (g/kg)	166.63	25.47	89.85	43.14	48.01
Soil total nitrogen content (g/kg)	2.37	0.27	0.97	0.46	47.6
Soil total phosphorus content (g/kg)	0.16	0.09	0.13	0.01	10.99

Abbreviations: CV, coefficient of variation; SD, standard deviation.

From the results of fruit setting ability and fruit and seed traits of *L. tsingtauense* (Table 2), the coefficient of variation of each index of fruit setting ability of *L. tsingtauense* was more than 30%. Except for the number of plump seeds per fruit, the coefficient of variation of fruit traits was less than 20%. The coefficient of variation of seed traits was 8.25% of thousand‐grain weight and 24.62% of seed germination percentage. It shows that the seed setting ability of *L. tsingtauense* is obviously different in different habitats. Except for the number of plump seeds per fruit, the variation of fruit traits was not large, and the traits were relatively stable. The variation range of seed germination percentage in seed traits was larger than that of thousand‐grain weight, and the difference was relatively obvious.

**TABLE 2:** Statistics on fruiting ability and seed and fruit traits of *L. tsingtauense*.

Index	Maximum	Minimum	Mean	SD	CV (%)
Fruiting ability					
Number of fruits	265	4	35.95	46.43	129.17
Large fruit rate (%)	0.54	0	0.25	0.15	60.11
Medium fruit rate (%)	0.83	0.14	0.48	0.16	34.2
Small fruit rate (%)	0.6	0	0.27	0.1	37.64
Fruit traits					
Fruit length (mm)	31.14	23.83	28.04	1.97	7.01
Fruit width (mm)	29.28	17.31	24.32	2.28	9.39
Fruit shape index	1.47	1	1.16	0.1	9.05
Number of plump seeds per fruit	48.55	10.33	31.97	8.51	26.61
Number of unfilled seeds per fruit	81.94	34.85	59.07	9.72	16.46
Number of seeds per fruit	119.84	63.2	91.04	11.74	12.9
Seed traits					
Thousand grain weight (g)	22.83	15.9	18.53	1.58	8.52
Germination percentage (%)	94	39.3	66.64	16.41	24.62

Abbreviations: CV, coefficient of variation; SD, standard deviation.

**FIGURE 3:**
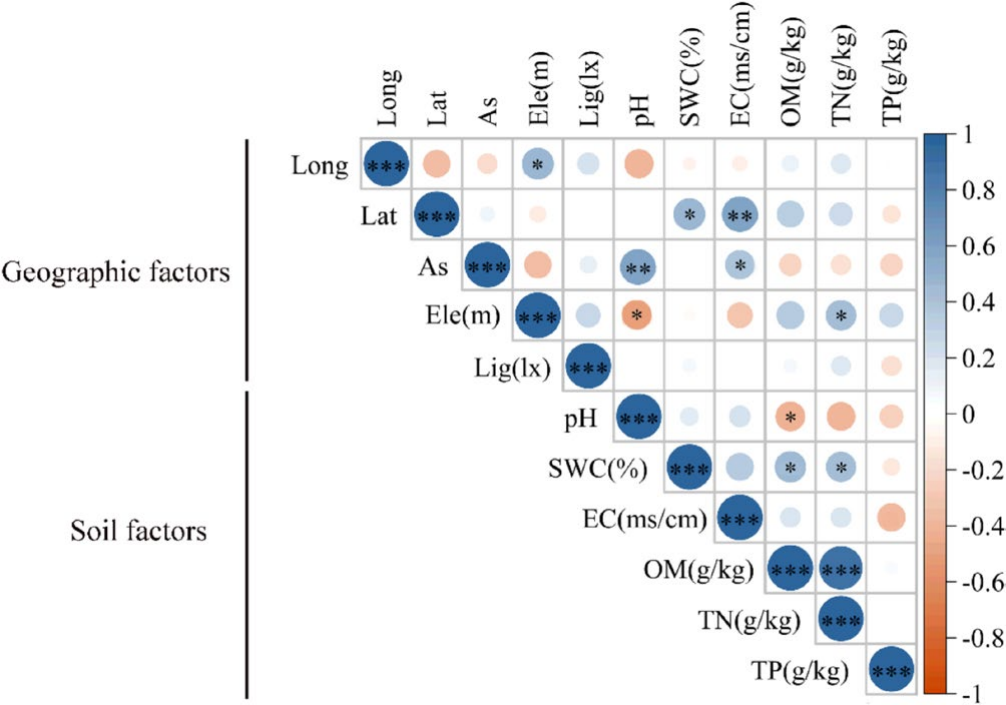
Correlation analysis between environmental factors of *Lilium tsingtauense*. Asterisks indicate the significance level after FDR correction among environmental factors: *q < 0.05, **q < 0.01, ***q < 0.001. Geographical factors (As, Aspect; Ele, Elevation; Lat, Latitude; Lig, Light intensity; Long, Longitude); Soil factors (EC, Soil Electrical Conductivity; OM, Soil Organic Matter Content; pH, Soil pH; SWC, Soil Water Content; TN, Soil Total Nitrogen Content; TP, Soil Total Phosphorus Content).

**FIGURE 4:**
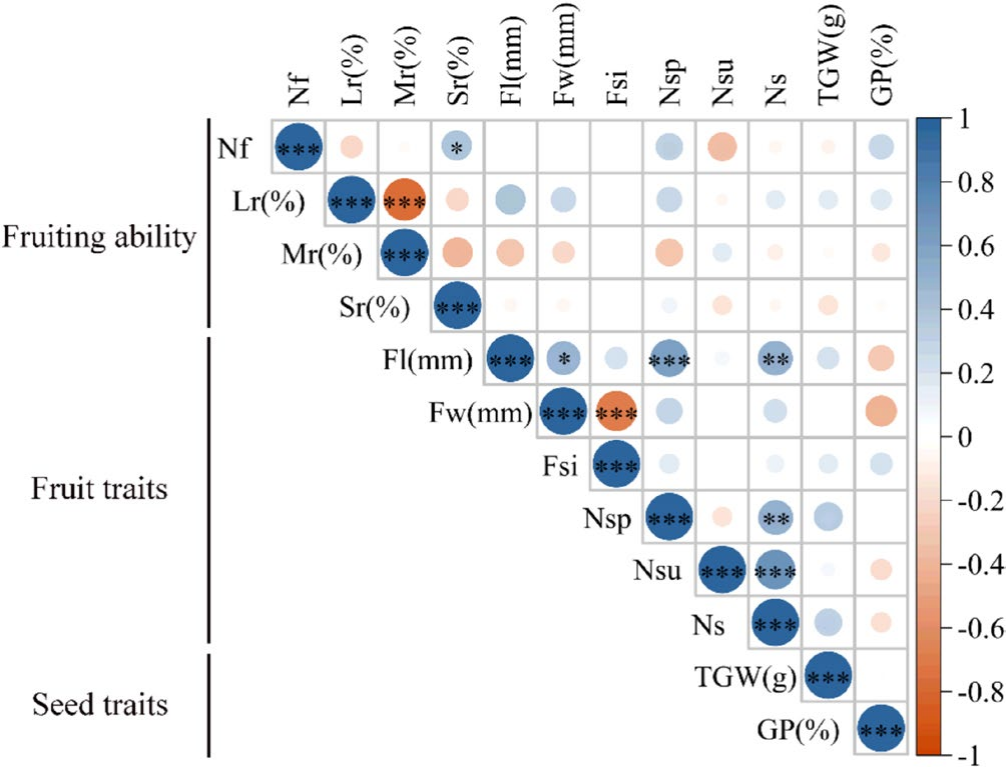
Correlation analysis between seed and fruit traits of *Lilium tsingtauense*. Asterisks indicate the significance level after FDR correction among fruit and seed traits: *q < 0.05, **q < 0.01, ***q < 0.001. Fruiting capacity (Lr, Large‐fruit rate; Mr., Medium‐fruit rate; Nf, Number of fruits; Sr., Small‐fruit rate); Fruit traits (Fl, Fruit length; Fsi, Fruit shape index; Fw, Fruit width; Ns, Number of seeds per fruit; Nsp, Number of plump seeds per fruit; Nsu, Number of unfilled seeds per fruit); Seed traits (GP, Germination percentage; TGW, Thousand grain weight).

**FIGURE 5:**
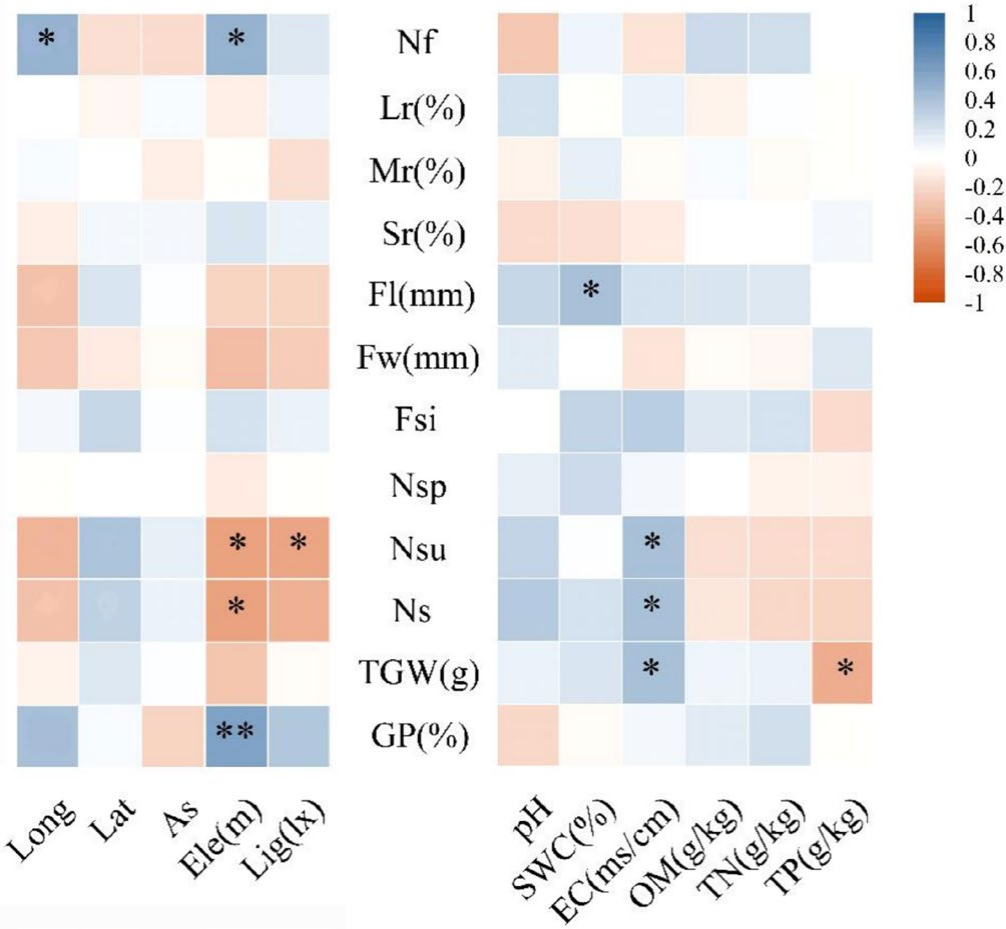
Correlation analysis between environmental factors and seed and fruit traits of *Lilium tsingtauense*. Asterisks indicate the significance level after FDR correction between environmental factors and seed and fruit traits: *q < 0.05, **q < 0.01. As, Aspect; EC, Soil Electrical Conductivity; Ele, Elevation; Fl, Fruit length; Fsi, Fruit shape index; Fw, Fruit width; GP, Germination percentage; Lat, Latitude; Lig, Light intensity; Long, Longitude; Lr, Large‐fruit rate; Mr., Medium‐fruit rate; Nf, Number of fruits; Ns, Number of seeds per fruit; Nsp, Number of plump seeds per fruit; Nsu, Number of unfilled seeds per fruit; OM, Soil Organic Matter Content; pH, Soil pH; Sr., Small‐fruit rate; SWC, Soil Water Content; TGW, Thousand grain weight; TN, Soil Total Nitrogen Content; TP, Soil Total Phosphorus Content.

Pearson correlation coefficients were employed to analyze the correlations among environmental factors of *L. tsingtauense* (Figure 3), among fruit and seed traits (Figure 4), and between environmental factors and seed and fruit traits (Figure 5). In the correlation analysis among environmental factors (Figure 3), longitude showed a significant positive correlation with elevation (FDR *q* < 0.05) but was not correlated with other environmental factors. Latitude exhibited highly significant positive correlations with soil water content (FDR *q* < 0.05) and electrical conductivity (FDR *q* < 0.01) among soil factors. Aspect was significantly positively correlated with soil pH (FDR *q* < 0.01) and electrical conductivity (FDR *q* < 0.05). Elevation showed a significant negative correlation with soil pH (FDR *q* < 0.05) and a significant positive correlation with soil total nitrogen content (FDR *q* < 0.05). Among soil factors, soil pH was significantly negatively correlated with soil organic matter content (FDR *q* < 0.05). Soil water content demonstrated highly significant positive correlations with soil organic matter content (FDR *q* < 0.05), total nitrogen content (FDR *q* < 0.05), as well as between soil organic matter content and total nitrogen content (FDR *q* < 0.001). Light intensity showed no correlation with other environmental factors.

In the analysis of the fruiting ability, fruit, and seed traits of *L. tsingtauense* (Figure 4), a significant positive correlation was found between fruit number and the small‐fruit rate (FDR *q* < 0.05), while a highly significant negative correlation was found between the large‐fruit rate and the medium‐fruit rate (FDR *q* < 0.001). Fruit length showed a significant positive correlation with fruit width (FDR *q* < 0.05), the number of plump seeds per fruit (FDR *q* < 0.001), and the number of seeds per fruit (FDR *q* < 0.01); a highly significant negative correlation was observed between fruit width and fruit shape index (FDR *q* < 0.001); the number of seeds per fruit was significantly positively correlated with the number of plump seeds per fruit (FDR *q* < 0.01) and the number of unfilled seeds per fruit (FDR *q* < 0.001).

In the correlation analysis between environmental factors and seed and fruit traits (Figure 5), fruit number showed significant positive correlations with longitude (FDR *q* < 0.05) and elevation (FDR *q* < 0.05). Fruit length was significantly positively correlated with soil water content (FDR *q* < 0.05). The number of unfilled seeds per fruit was significantly negatively correlated with elevation (FDR *q* < 0.05) and light intensity (FDR *q* < 0.05), but significantly positively correlated with soil electrical conductivity (FDR *q* < 0.05); the number of seeds per fruit was significantly negatively correlated with elevation (FDR *q* < 0.05) and significantly positively correlated with soil electrical conductivity (FDR *q* < 0.05). The thousand‐grain weight exhibited a significant positive correlation with soil electrical conductivity (FDR *q* < 0.05) but a significant negative correlation with soil total phosphorus content (FDR *q* < 0.05). Germination percentage demonstrated a highly significant positive correlation with elevation (FDR *q* < 0.01), while no correlation was found with any soil factors. No significant correlations were observed between the remaining fruiting ability or other seed and fruit traits and the environmental factors.

### 3.2 Redundancy Analysis of Seed and Fruit Traits and Environmental Factors of *L. tsingtauense*

DCA analysis was performed on the data (Schedule S1), and the model suitable for the specific data was judged according to the gradient length value of the first axis Axis1 of the analysis results. Axis1 < 3.0 indicates that the data is suitable for RDA of linear models. In this paper, environmental factors were used as explanatory variables (red arrows), and fruit and seed traits of *L. tsingtauense* were used as response variables (blue arrows) of environmental factors. The relationship between environmental factors and fruit and seed traits of *L. tsingtauense* was analyzed by linear constrained RDA ranking. Through the pre‐selection of environmental factors, the two environmental factors with the lowest interpretation of seed and fruit traits, namely aspect and soil total nitrogen content, were eliminated. The order of the interpretation rate of the remaining environmental factors is shown in Schedule S3.

**FIGURE 6:**
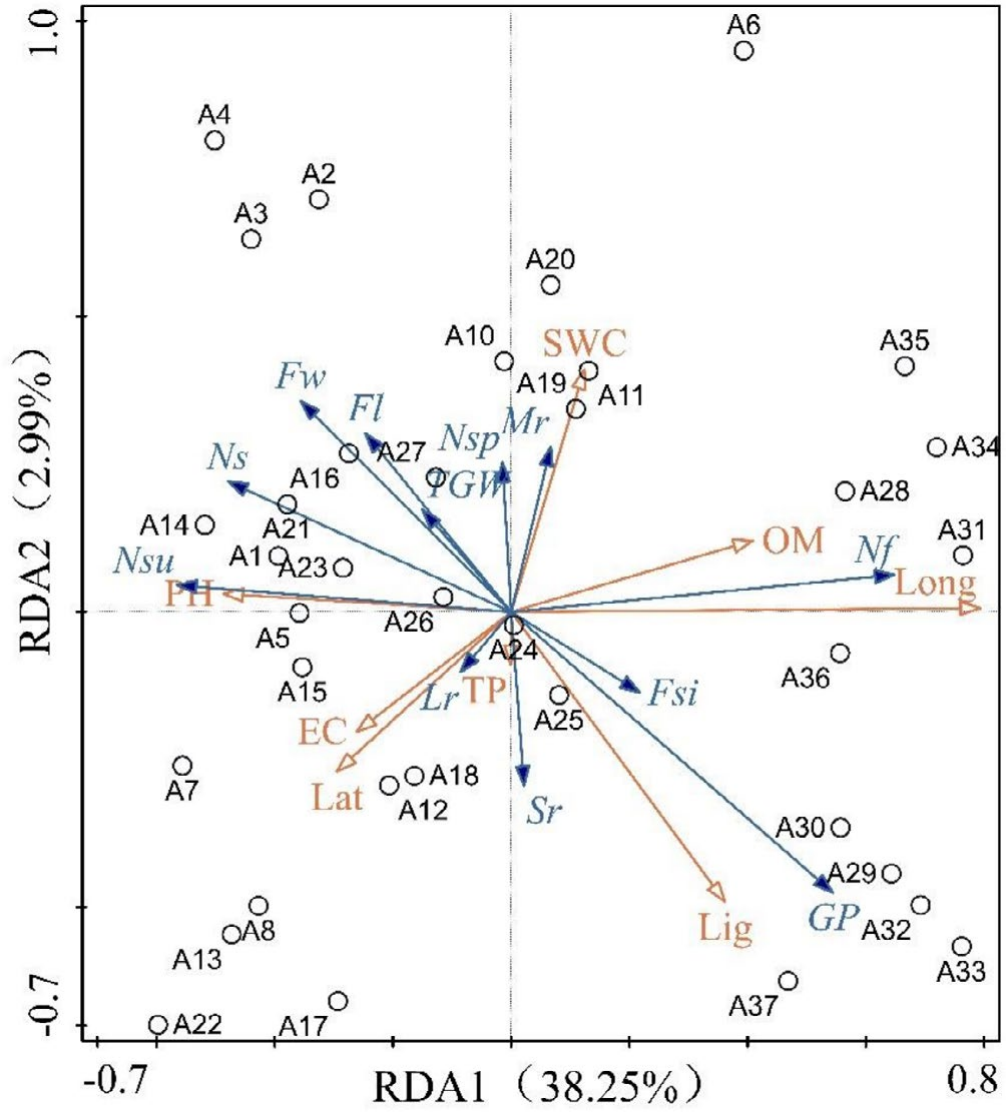
Redundancy analysis of seed and fruit traits and environmental factors of *Lilium tsingtauense*. A1‐37 represents 37 plots. Explanatory variable (EC, Soil Electrical Conductivity; Ele, Elevation; Lat, Latitude; Lig, Light intensity; Long, Longitude; OM, Soil Organic Matter Content; pH, Soil pH; SWC, Soil Water Content; TP, Soil Total Phosphorus Content); response variable (Fl, Fruit length; Fsi, Fruit shape index; Fw, Fruit width; GP, Germination percentage; Lr, Large‐fruit rate; Mr., Medium‐fruit rate; Nf, Number of fruits; Ns, Number of seeds per fruit; Nsp, Number of plump seeds per fruit; Nsu, Number of unfilled seeds per fruit; Sr., Small‐fruit rate; TGW, Thousand grain weight).

The distribution of plots in the RDA plot was scattered, indicating that the difference between different plots was significant (Figure 6). According to the results of the ranking chart (Schedule S2), the eigenvalues of the RDA1 axis and the RDA2 axis were 0.3825 and 0.0299, respectively. The cumulative interpretation rate was 41.24%, and the overall interpretation rate was 44.01%. The difference interpretation rates of RDA1 axis and RDA2 axis were 86.82% and 6.78%, respectively, and the correlation coefficients of seed and fruit traits and environmental factors were 0.7104 and 0.4751, respectively, indicating that the ranking results were reliable and representative, and could better characterize the correlation between seed and fruit traits and environmental factors. Compared with RDA2 axis, RDA1 axis could determine the correlation between fruit, seed traits and environmental factors of *L. tsingtauense*. According to the ranking table of environmental factor contribution rate (Schedule S3), Elevation (Ele) and longitude (Long) explained 26.5% (*p* < 0.05) and 7.9% (*p* < 0.05) of the variation of seed and fruit traits of *L. tsingtauense*, respectively, which were the potential key environmental factors affecting the difference of seed and fruit traits of *L. tsingtauense*.

### 3.3 Stepwise Linear Regression Analysis of Seed and Fruit Traits and Environmental Factors of *L. tsingtauense*

**FIGURE 7:**
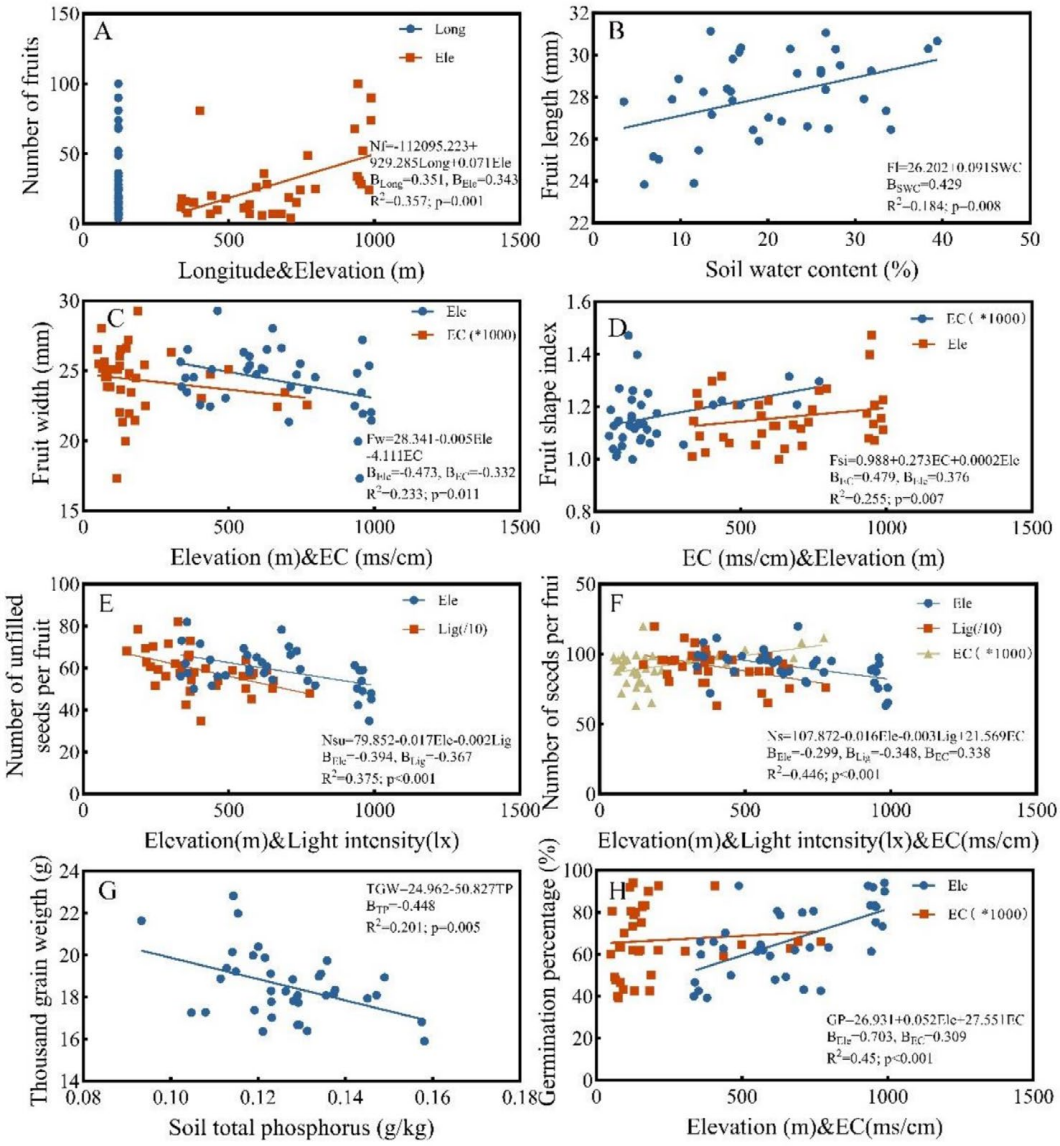
Stepwise linear regression analysis of seed and fruit traits and environmental factors of *Lilium tsingtauense*. (A) Number of fruits; (B) Fruit length; (C) Fruit width; (D) Fruit shape index; (E) Number of unfilled seeds per fruit; (F) Number of seeds per fruit; (G) Thousand grain weight; (H) Germination percentage.

In Figure 7 and Schedule S4, the regression equation of fruit number and environmental factors (Figure 7A) was significantly positively correlated with longitude and elevation. The standardized regression coefficient of longitude (0.351) was greater than that of elevation (0.343); that is, the potential environmental factors affecting fruit number were longitude, followed by elevation. In the regression equation of fruit length and environmental factors (Figure 7B), there was a significant positive correlation with soil water content; that is, the potential environmental factors affecting fruit length were soil water content. By analogy, fruit width was significantly negatively correlated with elevation and soil electrical conductivity, and the potential environmental factors were elevation, followed by soil electrical conductivity (Figure 7C). Fruit shape index was significantly positively correlated with soil electrical conductivity and elevation, and the potential environmental factors were soil electrical conductivity, followed by elevation (Figure 7D). The number of unfilled seeds per fruit showed a highly significant negative correlation with elevation and light intensity, with elevation being the potential key environmental factor influencing this trait, followed by light intensity (Figure 7E). The number of seeds per fruit exhibited a highly significant negative correlation with both elevation and light intensity, and a significant positive correlation with soil electrical conductivity; the potential key environmental factors influencing this trait were elevation, followed by light intensity and soil electrical conductivity (Figure 7F). There was a significant negative correlation between thousand grain weight and soil total phosphorus content, and the potential environmental factors were soil total phosphorus content (Figure 7G). Germination percentage was significantly positively correlated with elevation and soil electrical conductivity, and the potential environmental factors were elevation, followed by soil electrical conductivity (Figure 7H).

## 4 Discussion

The variation of fruit and seed traits is mainly determined by genetic factors, and is also affected by environmental factors such as climate variables (Diawara et al. 2025), Seed germination is the basis of natural regeneration of plant populations (Liu, Xiao, et al. 2024; Liu, Yang, et al. 2024). Relevant research is of great significance for plant population renewal and protection. In this paper, the differences in fruiting ability, fruit and seed traits of Chinese rare wild plant *L. tsingtauense* under different growth environments were investigated and analyzed. The study found that the number of fruits, the number of seeds per fruit, and the germination percentage of *L. tsingtauense* exhibited substantial variation, with significant differences observed across distinct habitats.; The number of fruits, fruit length, fruit width, fruit shape index, thousand grain weight and germination percentage were mainly affected by environmental factors such as longitude, elevation and soil water content; The number of fruits and germination percentage increased with the increase of elevation, and elevation was the potential key environmental factor affecting seed setting and germination. In general, our results showed that the seed and fruit traits of *L. tsingtauense* were affected by environmental factors. The r‐selection strategy was adopted to adapt to environmental changes, and the potential key environmental factor leading to differences in seed and fruit traits was elevation. A total of 150,925 wild *L. tsingtauense* were found in our field survey results. The limited number of wild *L. tsingtauense* makes it face more risks in the context of global change. This study is the first study on the response of seed traits to environmental changes in different habitats of *L. tsingtauense*, which has important implications for the protection and domestication of wild resources of *L. tsingtauense*.

### 4.1 Difference Analysis of Habitat Factors in Different Populations

The distribution area of *L. tsingtauense* is extremely narrow, most of which are distributed in Shandong Province of China, especially in the Laoshan Nature Reserve of Qingdao City. This study is carried out in this range. The coefficient of variation (CV) is a recognized method for standardizing the variability between factors with different units (Acasuso‐Rivero et al. 2019). In this study, except for longitude and latitude, the coefficient of variation of other environmental factors was 6.23%–90.93%, indicating that the environmental factors of the selected sample points were quite different. Through Pearson correlation analysis of environmental factors, it is found that there is a significant correlation between elevation and longitude in geographical factors, indicating that the selected sample points have changes in the geographical orientation of the region, showing the terrain characteristics of high in the east and low in the west, which is consistent with the terrain of Laoshan Mountain (Zhu et al. 2024), in line with the actual terrain of the study area.

The hydrothermal conditions brought by the increase of elevation promoted the growth of vegetation and the source of organic matter. At the same time, soil pH, slope aspect, and water conditions affect the decomposition and accumulation of organic matter. The interaction of these factors ultimately determines the content of soil organic matter and nitrogen (Cheng et al. 2006; Jego et al. 2023; Whitson 2015; Xue et al. 2012; Zhang et al. 2009). In this study, there is a significant correlation between geographical factors and soil factors, which is consistent with the above research results, indicating that various environmental factors interact and are closely related. The correlation analysis of environmental factors can understand the correlation between environmental factors and can play a supplementary role in the subsequent discussion of the relationship between plant traits and environmental factors and further analyze the mechanism of influencing factors.

### 4.2 Adaptation Strategies of Seed Traits of *L. tsingtauense*

Plant traits involve the response of the shape and structure of plant organs to environmental changes and the adaptation of plants to environmental heterogeneity (Sander et al. 2020). The coefficient of variation (CV) of phenotypic traits reflects the degree of dispersion of these traits. The CV value is usually considered to be the main indicator of variability. The lower the CV value, the smaller the dispersion of the trait (Zhang et al. 2024). For example, if the CV of a specific trait is greater than 10%, it indicates that the trait is significantly different between different varieties (Pélabon et al. 2020). In the study of different populations of *L. tsingtauense*, the CV value between fruit and seed traits was greater than 10%, which indicated that the difference between traits was significant, and it was more sensitive to the change of environmental factors and more susceptible to the influence of environmental changes. Seed germination is a key stage in the life cycle of plants, which determines the growth and survival of plants, and ultimately affects the composition of plant communities (Anniwaer et al. 2020). In this study, the variability of seed germination rate was 24.62%, which indicated that different environmental factors had a great influence on seed germination conditions, thus affecting the growth and development process of *L. tsingtauense* in the future.

The correlation between plant functional traits reflects the ecological regulation strategies adopted to cope with changes in environmental conditions (Kang et al. 2021). In plant phenotypes, the characteristics of plant fruits and seeds are an important feature in plant life history (Sander et al. 2020), it is very important for the formation of uniform seed batches, propagation research and production of high quality seedlings (Alle et al. 2024). There is a trade‐off among seed setting rate, seed size and number under the condition of limited total resources per plant (Chen et al. 2018). In this study, the correlation analysis of the fruiting ability, fruit and seed traits of *L. tsingtauense* found that there was a positive correlation between the number of fruits and the small fruit rate. The results of this study showed that among the fruits of *L. tsingtauense* sampled in the survey, small fruits accounted for more; that is, the fruits and seeds produced by *L. tsingtauense* in its growth environment were small. Seed size reflects the resource input of parents to individual offspring, and also affects the way of seed transmission. It is a key trait affecting plant colonization and reproduction (Huang et al. 2023). Small seeds have the advantages of mass production, longer survival in the seed bank, faster germination and lower seed predation rate (Olejniczak et al. 2018). However, species with larger seeds usually have higher seedling survival ability and stronger intraspecific competition ability than those with smaller seeds (Hou et al. 2021). In this study, *L. tsingtauense* produced smaller seeds and adopted an r‐reproduction strategy in its growth environment, so that *L. tsingtauense* showed high reproductive capacity but low survival rate of larvae. However, in the face of changing environment, seedling plants are difficult to survive, which may be one of the reasons for the endangerment of *L. tsingtauense*.

### 4.3 Environmental Factors Affecting the Seed and Fruit Traits of *L. tsingtauense*

Different species exhibit varying sensitivity and adaptability to their environments. Intraspecific variation reflects the adaptation of populations to diverse environmental conditions, and therefore, it can serve, to a certain extent, as an indicator of a species' adaptability to complex environments (De Kort et al. 2021). Seed traits play a crucial role in understanding plant community ecology, largely determining a plant's ability to disperse and re‐establish (Jiménez‐Alfaro et al. 2016); these traits are significantly influenced by geographical location, habitat, and year (Wang et al. 2022; Wu et al. 2018). Due to differences in habitat conditions such as elevation, climate, soil, biota, and topography across geographical locations, different populations of the same plant species, different individuals within the same population, and different years may exhibit not only variations in seed set but also significant differences in seed size under natural conditions (Alcantara and Rey 2003).

Integrated with Pearson correlation analysis, RDA, and stepwise linear regression analysis, it was found that, compared to other environmental factors, the number of fruits, the number of unfilled seeds per fruit, the number of seeds per fruit, and the germination percentage were closely related to elevation, identifying elevation as a potential key environmental factor influencing these traits. Elevation can lead to variations in temperature and moisture, thereby affecting species' germination and regeneration strategies (Oda et al. 2016). The observed traits may represent adaptive adjustments of *L. tsingtauense* to the environment of Laoshan Mountain. In our study, the number of fruits and the germination percentage increased with rising elevation, whereas the number of unfilled seeds per fruit and the number of seeds per fruit decreased with increasing elevation. This finding suggests that *L. tsingtauense* produces a greater number of fruits in high‐elevation habitats while ensuring that its seeds are plump and exhibit a high germination rate, prioritizing quality in their energy investment. Variation in seed traits, particularly seed mass, is often associated with environmental conditions such as climate, soil nutrients, and moisture (Bhatt et al. 2020). The interactions between plants and their environment form a complex and interdependent system (Li et al. 2024). Soil serves as the primary medium for plant growth, supplying essential nutrients and water while influencing root development and overall plant health (Wong et al. 2024). Litter accumulation, organic matter content, and soil acidity account for variations in components such as temperature and moisture along elevational gradients (De Feudis et al. 2025; Liao et al. 2024). In this study, elevation was closely correlated with soil pH, soil organic matter, and soil total nitrogen content. This result may be attributed to the rich vegetation diversity, humid understory conditions, and thick litter accumulation in the high‐elevation areas of Laoshan Mountain, which indirectly ensure an adequate nutrient supply for *L. tsingtauense* during growth, thereby influencing seed mass. This interpretation is supported by studies investigating the relationship between seed mass and environmental factors (Bazzato et al. 2021; Long et al. 2025).

In this study, the lack of significant correlation between most fruit and seed traits and the majority of environmental factors, as well as the varying degrees of correlation between different fruit and seed traits and environmental factors, may be attributed to the relatively low phenotypic plasticity of reproductive traits such as seed and fruit characteristics (Sukhorukov et al. 2023). Due to trait differences, they exhibit relatively independent environmental adaptations when facing different selective pressures (Zhao et al. 2026). Stepwise linear regression analysis revealed that fruit width, fruit shape index, number of seeds per fruit, and germination percentage were all closely related to elevation and soil electrical conductivity, further indicating that elevation and soil factors influence plant growth. This may be attributed to the interaction between soil nutrients and meteorological factors under different elevation conditions, which in turn affects seed traits (Long et al. 2025). Stepwise linear regression analysis also revealed that fruit number was closely related to longitude and elevation. In addition to environmental factors, human activities may also influence fruit number; in low‐elevation areas, frequent human activity raises the possibility of anthropogenic collection. Therefore, when conserving wild *L. tsingtauense* resources, particular attention should be paid to whether plants in low‐elevation areas are damaged. Conservation awareness initiatives for rare plants should be further strengthened to provide a safe environmental range for their growth and reproduction.

## 5 Conclusion

In summary, this study analyzed the fruit and seed traits of different populations of *L. tsingtauense*, and explored the association between these traits and the effects of environmental factors on these traits. The results showed that there were significant differences in fruit and seed traits under different growth environment conditions, and the environmental factors had different effects on different traits. Among them, the number of fruits and seed germination rate are greatly affected by altitude factors. As a rare wild plant, *Lilium tsingtauense* offers valuable insights for the regeneration and conservation of its wild resources through an analysis of the relationship between its seed and fruit traits and environmental factors. This study represents the first investigation into the responses of seed and fruit traits of *L. tsingtauense* populations from different habitats to environmental changes. Unlike previous studies focusing on individual growth and biomass accumulation, this research emphasizes the limiting effects of external environmental conditions—specifically, different geographical factors and soil environments—on the reproduction of *L. tsingtauense* by analyzing its seed and fruit traits. The results indicate that *L. tsingtauense* in high‐elevation areas produces a greater number of fruits and exhibits the highest germination rate, with elevation identified as a potential key environmental factor influencing seed and fruit traits. These findings provide a theoretical basis for the artificial regeneration, cultivation, and management of *L. tsingtauense*, thereby enhancing the efficiency of conservation efforts for its populations.

## Author Contributions

**Wanpei Lu:** conceptualization (equal), data curation (equal), formal analysis (equal), investigation (equal), methodology (equal), visualization (equal), writing – original draft (equal), writing – review and editing (equal). **Anning Ding:** data curation (equal), formal analysis (equal), investigation (equal), visualization (equal), writing – original draft (equal). **Xiao Guo:** conceptualization (equal), methodology (equal), visualization (equal), writing – review and editing (equal). **Pulin Sun:** data curation (equal), investigation (equal). **Xinqiang Jiang:** conceptualization (equal), investigation (equal), methodology (equal). **Jinming Yang:** investigation (equal). **Hai Wang:** investigation (equal). **Xuebin Song:** investigation (equal). **Qingchao Liu:** conceptualization (equal), funding acquisition (equal), investigation (equal), methodology (equal), project administration (equal), writing – original draft (equal), writing – review and editing (equal).

## Conflicts of Interest

The authors declare no conflicts of interest.

## Supporting information


**Appendix S1:** ece373238‐sup‐0001‐AppendixS1.docx.

## Data Availability

Data input files of fruit seed traits and environmental factors can be found on: https://doi.org/10.5061/dryad.sf7m0cgnh.

## References

[ece373238-bib-0001] Acasuso‐Rivero, C. , C. J. Murren , C. D. Schlichting , and U. K. Steiner . 2019. “Adaptive Phenotypic Plasticity for Life‐History and Less Fitness‐Related Traits.” Proceedings of the Royal Society B: Biological Sciences 286, no. 1904: 9. 10.1098/rspb.2019.0653. PMC6571476 31185861

[ece373238-bib-0003] Alcantara, J. M. , and P. J. Rey . 2003. “Conflicting Selection Pressures on Seed Size: Evolutionary Ecology of Fruit Size in a Bird‐Dispersed Tree, *Olea europaea* .” Journal of Evolutionary Biology 16, no. 6: 1168–1176. 10.1046/j.1420-9101.2003.00618.x. 14640408

[ece373238-bib-0004] Ali, S. , S. M. Khan , Z. Ahmad , et al. 2023. “Relative Humidity, Soil Phosphorus, and Stand Structure Diversity Determine Aboveground Biomass Along the Elevation Gradient in Various Forest Ecosystems of Pakistan.” Sustainability 15, no. 9: 16. 10.3390/su15097523.

[ece373238-bib-0005] Alle, T. R. , S. M. Andrew , M. F. Karlsson , and A. Gure . 2024. “Morphological Traits of Fruits and Seeds of Ziziphus Tree Species Growing in Different Land Uses in Ethiopia.” Heliyon 10, no. 14: 7. 10.1016/j.heliyon.2024.e34751. PMC11315178 39130437

[ece373238-bib-0006] Anniwaer, A. , Y. G. Su , X. B. Zhou , and Y. M. Zhang . 2020. “Impacts of Snow on Seed Germination Are Independent of Seed Traits and Plant Ecological Characteristics in a Temperate Desert of Central Asia.” Journal of Arid Land 12, no. 5: 775–790. 10.1007/s40333-020-0059-9.

[ece373238-bib-0007] Bachman, S. P. , M. J. M. Brown , T. C. C. Leao , E. N. Lughadha , and B. E. Walker . 2024. “Extinction Risk Predictions for the World's Flowering Plants to Support Their Conservation.” New Phytologist 242, no. 2: 797–808. 10.1111/nph.19592. 38437880

[ece373238-bib-0008] Bai, N. Y. , Y. J. Song , Y. Li , et al. 2024. “Evaluation of Five Asian Lily Cultivars in Chongqing Province China and Effects of Exogenous Substances on the Heat Resistance.” Horticulturae 10, no. 11: 16. 10.3390/horticulturae10111216.

[ece373238-bib-0009] Bazzato, E. , E. Serra , S. Maccherini , and M. Marignani . 2021. “Reduction of Inter‐ and Intraspecific Seed Mass Variability Along a Land‐Use Intensification Gradient.” Ecological Indicators 129: 10. 10.1016/j.ecolind.2021.107884.

[ece373238-bib-0010] Bhatt, A. , N. R. Bhat , A. Al‐Nasser , M. M. Carón , and A. Santo . 2020. “Inter‐Population Variabilities in Seed Mass and Germination of *Panicum turgidum* and *Pennisetum divisum* on the Desert of Kuwait.” Journal of Arid Land 12, no. 1: 144–153. 10.1007/s40333-019-0017-6.

[ece373238-bib-0011] Chen, K. , K. S. Burgess , X. Y. Yang , Y. H. Luo , L. M. Gao , and D. Z. Li . 2018. “Functional Trade‐Offs and the Phylogenetic Dispersion of Seed Traits in a Biodiversity Hotspot of the Mountains of Southwest China.” Ecology and Evolution 8, no. 4: 2218–2230. 10.1002/ece3.3805. 29468038 PMC5817125

[ece373238-bib-0012] Cheng, J. M. , H. E. Wan , X. M. Hu , and Y. Y. zhao . 2006. “Accumulation and Decomposition of Litter in the Semiarid Enclosed Grassland.” Acta Ecologica Sinica 26, no. 4: 1207–1212.

[ece373238-bib-0013] Chung, M. Y. , J. López‐Pujol , and M. G. Chung . 2014. “Comparative Biogeography of the Congener Lilies *Lilium distichum* and *Lilium tsingtauense* in Korea.” Flora 209, no. 8: 435–445. 10.1016/j.flora.2014.04.005.

[ece373238-bib-0014] Dawson, W. , J. Bòdis , A. Bucharova , et al. 2024. “Root Traits Vary as Much as Leaf Traits and Have Consistent Phenotypic Plasticity Among 14 Populations of a Globally Widespread Herb.” Functional Ecology 38, no. 4: 926–941. 10.1111/1365-2435.14504.

[ece373238-bib-0015] De Feudis, M. , L. Massaccesi , C. Poesio , L. V. Antisari , R. Bol , and A. Agnelli . 2025. “Seasonal Effects of Altitude and Vegetation on Litter and Organic Carbon in Deciduous and Coniferous Forest Soils.” Geoderma 459: 13. 10.1016/j.geoderma.2025.117382.

[ece373238-bib-0016] De Kort, H. , J. G. Prunier , S. Ducatez , et al. 2021. “Life History, Climate and Biogeography Interactively Affect Worldwide Genetic Diversity of Plant and Animal Populations.” Nature Communications 12, no. 1: 11. 10.1038/s41467-021-20958-2. PMC7822833 33483517

[ece373238-bib-0017] Diawara, S. , F. Bognounou , P. Savadogo , and A. Ouédraogo . 2025. “Variation in Fruits and Seeds Traits of *Saba senegalensis* (A. DC.) Pichon Along a Climatic Gradient in Burkina Faso, West Africa: Implications for Its Sustainable Management.” Genetic Resources and Crop Evolution 72, no. 3: 3525–3541. 10.1007/s10722-024-02178-x.

[ece373238-bib-0018] Duan, G. H. , Z. M. Wen , W. Xue , et al. 2022. “Agents Affecting the Plant Functional Traits in National Soil and Water Conservation Demonstration Park (China).” Plants (Basel) 11, no. 21: 16. 10.3390/plants11212891. PMC9657439 36365344

[ece373238-bib-0019] Famiglietti, C. A. , M. Worden , L. D. L. Anderegg , and A. G. Konings . 2024. “Impacts of Climate Timescale on the Stability of Trait‐Environment Relationships.” New Phytologist 241, no. 6: 2423–2434. 10.1111/nph.19416. 38037289

[ece373238-bib-0020] Fang, D. Y. , X. Y. Zhao , Y. Q. Pan , et al. 2025. “Screening Criteria for High‐Quality *Glycyrrhiza uralensis* Fisch. Seeds From the Ordos Plateau.” Scientific Reports 15, no. 1: 12. 10.1038/s41598-025-05736-0. 40596035 PMC12216282

[ece373238-bib-0021] Fox, R. J. , J. M. Donelson , C. Schunter , T. Ravasi , and J. D. Gaitán‐Espitia . 2019. “Beyond Buying Time: The Role of Plasticity in Phenotypic Adaptation to Rapid Environmental Change.” Philosophical Transactions of the Royal Society, B: Biological Sciences 374, no. 1768: 9. 10.1098/rstb.2018.0174. PMC6365870 30966962

[ece373238-bib-0024] Gong, H. M. , M. Yang , C. C. Wang , and C. L. Tian . 2023. “Leaf Phenotypic Variation and Its Response to Environmental Factors in Natural Populations of *Eucommia ulmoides* .” BMC Plant Biology 23, no. 1: 14. 10.1186/s12870-023-04583-3. 37964219 PMC10647038

[ece373238-bib-0025] Guo, M. F. , J. Zong , J. X. Zhang , et al. 2024. “Effects of Temperature and Drought Stress on the Seed Germination of a Peatland Lily (*Lilium concolor* Var. *Megalanthum*).” Frontiers in Plant Science 15: 11. 10.3389/fpls.2024.1462655. PMC11514071 39469053

[ece373238-bib-0026] Guo, W. H. , J. Jeong , Z. Kim , R. Q. Wang , E. Kim , and S. Kim . 2011. “Genetic Diversity of Lilium Tsingtauense in China and Korea Revealed by ISSR Markers and Morphological Characters.” Biochemical Systematics and Ecology 39, no. 4–6: 352–360. 10.1016/j.bse.2011.05.002.

[ece373238-bib-0027] Guo, X. , H. J. Song , P. Wu , et al. 2025. “Intraspecific Elementome Variation of the Clonal Grass *Phragmites australis* Reflects Environmental Variation More Than Genetic and Epigenetic Variation.” Journal of Plant Ecology 18, no. 4: 15. 10.1093/jpe/rtaf070.

[ece373238-bib-0028] Guo, X. , Z. H. Sun , Y. F. Gao , et al. 2025. “Haplotype‐Specific Interactions of *Phragmites australis* With *Spartina alterniflora* Under Salt Stress.” Journal of Environmental Management 384: 11. 10.1016/j.jenvman.2025.125506. 40294447

[ece373238-bib-0029] He, N. P. , Y. Li , C. C. Liu , et al. 2020. “Plant Trait Networks: Improved Resolution of the Dimensionality of Adaptation.” Trends in Ecology & Evolution 35, no. 10: 908–918. 10.1016/j.tree.2020.06.003. 32595068

[ece373238-bib-0030] Hou, J. F. , F. Li , Z. H. Wang , X. Q. Li , R. Cao , and W. Q. Yang . 2023. “Seasonal Dynamics of Sediment Organic Carbon Storage for the Upper Streams of the Yangtze River.” Frontiers in Ecology and Evolution 11: 10. 10.3389/fevo.2023.1093448.

[ece373238-bib-0031] Hou, J. W. , Z. B. Nan , C. Baskin , and T. Chen . 2021. “Effect of Seed Size and Fungicide on Germination and Survival of Buried Seeds of Two Grassland Species on the Loess Plateau, China.” Acta Oecologica 110: 6. 10.1016/j.actao.2021.103716.

[ece373238-bib-0032] Huang, C. S. , Y. Xu , and R. G. Zang . 2021. “Variation Patterns of Functional Trait Moments Along Geographical Gradients and Their Environmental Determinants in the Subtropical Evergreen Broadleaved Forests.” Frontiers in Plant Science 12: 12. 10.3389/fpls.2021.686965. PMC8311185 34322143

[ece373238-bib-0033] Huang, L. J. , Z. K. Zhang , Q. Wang , G. M. Yang , Q. M. Que , and X. Z. Liu . 2023. “Impact of Landscape Patterns on Herb‐Layer Diversity and Seed Size of *Schima superba* in Urban Remnant Vegetation: A Case Study in Guangzhou, Southern China.” Tropical Ecology 64, no. 3: 452–463. 10.1007/s42965-022-00255-9.

[ece373238-bib-0034] Huang, W. W. , E. Olson , S. C. Wang , and P. J. Shi . 2020. “The Growth and Mortality of Pleioblastus Pygmaeus Under Different Light Availability.” Global Ecology and Conservation 24: 14. 10.1016/j.gecco.2020.e01262.

[ece373238-bib-0036] Jego, L. , R. N. Li , S. Roudine , et al. 2023. “Parasitoid Ecology Along Geographic Gradients: Lessons for Climate Change Studies.” Current Opinion in Insect Science 57: 8. 10.1016/j.cois.2023.101036. 37061184

[ece373238-bib-0037] Jiménez‐Alfaro, B. , F. A. O. Silveira , A. Fidelis , P. Poschlod , and L. E. Commander . 2016. “Seed Germination Traits Can Contribute Better to Plant Community Ecology.” Journal of Vegetation Science 27, no. 3: 637–645. 10.1111/jvs.12375.

[ece373238-bib-0038] Kang, Y. I. , Y. J. Choi , Y. R. Lee , K. H. Seo , J. N. Suh , and H. R. Lee . 2021. “Cut Flower Characteristics and Growth Traits Under Salt Stress in Lily Cultivars.” Plants (Basel) 10, no. 7: 12. 10.3390/plants10071435. PMC8309348 34371643

[ece373238-bib-0039] Kerr, K. L. , J. C. Fickle , and W. R. L. Anderegg . 2023. “Decoupling of Functional Traits From Intraspecific Patterns of Growth and Drought Stress Resistance.” New Phytologist 239, no. 1: 174–188. 10.1111/nph.18937. 37129078

[ece373238-bib-0040] Klavina, D. , A. Osvalde , G. Tabors , and G. Jakobsone . 2025. “Current Status of *Pulsatilla patens* in Latvia‐Population Size, Demographic and Seed Viability Indicators, Soil Parameters and Their Relationships.” Plants (Basel) 14, no. 3: 17. 10.3390/plants14030375. PMC11819818 39942937

[ece373238-bib-0041] Lee, S. C. , K. Kim , Y. J. Hwang , K. B. Lim , and T. J. Yang . 2016. “The Complete Chloroplast Genomes of *Lilium tsingtauense* Gilg (Liliaceae).” Mitochondrial DNA Part B‐Resources 1, no. 1: 336–337. 10.1080/23802359.2016.1172052. 33644375 PMC7871828

[ece373238-bib-0042] Li, Q. , J. Y. Yang , G. X. He , X. N. Liu , and D. G. Zhang . 2022. “Characteristics of Soil C:N:P Stoichiometry and Enzyme Activities in Different Grassland Types in Qilian Mountain Nature Reserve‐Tibetan Plateau.” PLoS One 17, no. 7: 14. 10.1371/journal.pone.0271399. PMC9282613 35834549

[ece373238-bib-0043] Li, S. H. , G. Y. Gao , C. Wang , Z. S. Li , X. M. Feng , and B. J. Fu . 2024. “Aridity Regulates the Impacts of Multiple Dimensional Plant Diversity on Soil Properties in the Drylands of Northern China.” Science of the Total Environment 946: 9. 10.1016/j.scitotenv.2024.174211. 38914324

[ece373238-bib-0044] Liao, C. , K. K. Chang , B. Y. Wu , D. D. Zhang , C. Wang , and X. L. Cheng . 2024. “Divergence in Soil Particulate and Mineral‐Associated Organic Carbon Reshapes Carbon Stabilization Along an Elevational Gradient.” Catena 235: 9. 10.1016/j.catena.2023.107682.

[ece373238-bib-0045] Liao, G. X. , X. D. Ning , Y. L. Yang , et al. 2023. “Main Habitat Factors Driving the Phenotypic Diversity of *Litsea cubeba* in China.” Plants (Basel) 12, no. 21: 17. 10.3390/plants12213781. PMC10648399 37960137

[ece373238-bib-0046] Liu, B. D. , J. M. Yang , W. P. Lu , et al. 2024. “Altitudinal Variation in Rhizosphere Microbial Communities of the Endangered Plant *Lilium tsingtauense* and the Environmental Factors Driving This Variation.” Microbiology Spectrum 12, no. 11: 19. 10.1128/spectrum.00966-24. PMC11536999 39382299

[ece373238-bib-0047] Liu, R. T. , P. Han , J. Wang , et al. 2025. “Exploration and Empirical Study on Spatial Distribution of SOC at the Core Area in Coastal Tamarix Forests' Inland Side of Changyi National Marine Ecological Area.” Forests 16, no. 1: 15. 10.3390/f16010169.

[ece373238-bib-0048] Liu, X. S. , Y. F. Xiao , Y. M. Ling , et al. 2023. “Effects of Seed Biological Characteristics and Environmental Factors on Seed Germination of the Critically Endangered Species *Hopea chinensis* (Merr.) Hand.‐Mazz. In China.” Forests 14, no. 10: 17. 10.3390/f14101975.

[ece373238-bib-0049] Liu, X. S. , Y. F. Xiao , Y. Wang , et al. 2024. “Seed Germination Ecology of Endangered Plant *Horsfieldia hainanensis* Merr. In China.” BMC Plant Biology 24, no. 1: 17. 10.1186/s12870-024-05208-z. 38822268 PMC11143685

[ece373238-bib-0050] Long, J. T. , X. L. Gao , and Y. J. Miao . 2025. “Effects of Environmental Factors on the Phenotypic Traits and Seed Element Accumulation of Wild *Elymus nutans* in Tibet.” Scientific Reports 15, no. 1: 15. 10.1038/s41598-025-85415-2. 39805884 PMC11731037

[ece373238-bib-0052] Macrì, C. , D. Dagnino , M. Guerrina , et al. 2021. “Effects of Environmental Heterogeneity on Phenotypic Variation of the Endemic Plant *Lilium pomponium* in the Maritime and Ligurian Alps.” Oecologia 195, no. 1: 93–103. 10.1007/s00442-020-04806-6. 33269409 PMC7882563

[ece373238-bib-0053] Oda, G. A. M. , M. I. G. Braz , and R. d. Q. Portela . 2016. “Does Regenerative Strategy Vary Between Populations? A Test Using a Narrowly Distributed Atlantic Rainforest Palm Species.” Plant Ecology 217, no. 7: 869–881. 10.1007/s11258-016-0612-y.

[ece373238-bib-0054] Olejniczak, P. , M. Czarnoleski , A. Delimat , B. M. Majcher , and K. Szczepka . 2018. “Seed Size in Mountain Herbaceous Plants Changes With Elevation in a Species‐Specific Manner.” PLoS One 13, no. 6: 14. 10.1371/journal.pone.0199224. PMC6005539 29912939

[ece373238-bib-0056] Paz‐Dyderska, S. , and A. M. Jagodzinski . 2024. “Potential of Reproductive Traits in Functional Ecology: A Quantitative Comparison of Variability in Floral, Fruit, and Leaf Traits.” Ecology and Evolution 14, no. 7: 17. 10.1002/ece3.11690. PMC11255459 39026952

[ece373238-bib-0057] Pélabon, C. , C. H. Hilde , S. Einum , and M. Gamelon . 2020. “On the Use of the Coefficient of Variation to Quantify and Compare Trait Variation.” Evolution Letters 4, no. 3: 180–188. 10.1002/evl3.171. 32547779 PMC7293077

[ece373238-bib-0058] Pritzkow, C. , V. Williamson , C. Szota , R. Trouvé , and S. K. Arndt . 2020. “Phenotypic Plasticity and Genetic Adaptation of Functional Traits Influences Intra‐Specific Variation in Hydraulic Efficiency and Safety.” Tree Physiology 40, no. 2: 215–229. 10.1093/treephys/tpz121. 31860729

[ece373238-bib-0059] Qiu, D. , Y. Zhang , X. D. Ma , et al. 2026. “Altitude Markedly Influenced Moss Functional Traits and Trait Associations at the Community Level in the Eastern Pamir Plateau.” Environmental and Experimental Botany 241: 16. 10.1016/j.envexpbot.2025.106293.

[ece373238-bib-0060] Ren, L. J. , X. Guo , B. K. Sorrell , F. Eller , and H. Brix . 2025. “Responses to Cold Temperature Determine Clinal Patterns of Photosynthetic Acclimation of a Cosmopolitan Grass Genus and Challenge the Concept of Quantifying Phenotypic Plasticity.” Functional Ecology 39, no. 2: 583–595. 10.1111/1365-2435.14734.

[ece373238-bib-0061] Ribeiro, G. V. T. , A. L. Teixido , N. P. U. Barbosa , and F. A. O. Silveira . 2016. “Assessing Bias and Knowledge Gaps on Seed Ecology Research: Implications for Conservation Agenda and Policy.” Ecological Applications 26, no. 7: 2033–2043. 10.1890/15-1852.1. 27755716

[ece373238-bib-0062] Rojas, T. N. , I. C. Zampini , M. I. Isla , and P. G. Blendinger . 2022. “Fleshy Fruit Traits and Seed Dispersers: Which Traits Define Syndromes?” Annals of Botany 129, no. 7: 831–838. 10.1093/aob/mcab150. 34918034 PMC9292605

[ece373238-bib-0064] Rosin, C. , and J. R. Poulsen . 2018. “Seed Traits, Not Density or Distance From Parent, Determine Seed Predation and Establishment in an Afrotropical Forest.” Biotropica 50, no. 6: 881–888. 10.1111/btp.12601.

[ece373238-bib-0065] Sander, N. L. , C. J. da Silva , A. V. M. Duarte , et al. 2020. “The Influence of Environmental Features on the Morphometric Variation in *Mauritia flexuosa* L.f. Fruits and Seeds.” Plants 9, no. 10: 10. 10.3390/plants9101304. PMC7600886 33023147

[ece373238-bib-0066] Si, C. , F. Li , and W. Bo . 2022. “Seed Size Affects Rodent‐Seed Interaction Consistently Across Plant Species but Not Within Species: Evidence From a Seed Tracking Experiment of 41 Tree Species.” Integrative Zoology 17, no. 5: 930–943. 10.1111/1749-4877.12619. 34936198

[ece373238-bib-0068] Song, H. J. , X. Guo , J. C. Yang , et al. 2024. “Phenotypic Plasticity Variations in *Phragmites australis* Under Different Plant–Plant Interactions Influenced by Salinity.” Journal of Plant Ecology 17, no. 3: 9. 10.1093/jpe/rtae035.

[ece373238-bib-0069] Sukhorukov, A. P. , M. S. Sousa‐Baena , M. S. Romanov , and X. Wang . 2023. “Editorial: Fruit and Seed Evolution in Angiosperms.” Frontiers in Plant Science 14: 2. 10.3389/fpls.2023.1196443. PMC10154684 37152136

[ece373238-bib-0071] Tuthill, J. E. , Y. K. Ortega , and D. E. Pearson . 2023. “Seed Size, Seed Dispersal Traits, and Plant Dispersion Patterns for Native and Introduced Grassland Plants.” Plants (Basel) 12, no. 5: 12. 10.3390/plants12051032. PMC10005497 36903896

[ece373238-bib-0072] Urrutia‐Jalabert, R. , D. E. Carvajal , R. Skelton , et al. 2025. “Intraspecific Variation in Drought Tolerance Traits in a *Nothofagus* Species (Southern Beech) in Southern South America.” Forest Ecology and Management 597: 13. 10.1016/j.foreco.2025.123165.

[ece373238-bib-0073] Visscher, A. M. , F. Vandelook , E. Fernández‐Pascual , et al. 2022. “Low Availability of Functional Seed Trait Data From the Tropics Could Negatively Affect Global Macroecological Studies, Predictive Models and Plant Conservation.” Annals of Botany 130, no. 6: 773–784. 10.1093/aob/mcac130. 36349952 PMC9758304

[ece373238-bib-0074] Wang, H. Y. , C. Tong , J. F. Huang , et al. 2022. “Latitudinal Variations of Seed Functional Traits of *Phragmites australis* in Chinese Coastal Marsh.” Ying Yong Sheng Tai Xue Bao 33, no. 12: 3294–3302. 10.13287/j.1001-9332.202212.001. 36601834

[ece373238-bib-0075] Wang, M. L. , J. X. Zhang , Z. P. Guo , et al. 2020. “Morphological Variation in *Cynodon dactylon* (L.) Pers., and Its Relationship With the Environment Along a Longitudinal Gradient.” Hereditas 157, no. 1: 11. 10.1186/s41065-020-00117-1. 32051037 PMC7014724

[ece373238-bib-0076] Whitson, I. R. 2015. “Equivalent Latitude for Prediction of Soil Development in a Complex Mapunit.” Canadian Journal of Soil Science 95, no. 2: 125–137. 10.4141/cjss-2014-074.

[ece373238-bib-0077] Wong, W. S. , J. Ruscalleda‐Alvarez , J. W. H. Yong , J. C. Stevens , J. M. Valliere , and E. J. Veneklaas . 2024. “Limited Efficacy of a Commercial Microbial Inoculant for Improving Growth and Physiological Performance of Native Plant Species.” Conservation Physiology 12, no. 1: 19. 10.1093/conphys/coae037. PMC11184453 38894755

[ece373238-bib-0078] Wu, H. , H. J. Meng , S. T. Wang , X. Z. Wei , and M. X. Jiang . 2018. “Geographic Patterns and Environmental Drivers of Seed Traits of a Relict Tree Species.” Forest Ecology and Management 422: 59–68. 10.1016/j.foreco.2018.04.003.

[ece373238-bib-0079] Wu, Y. P. , M. J. Ding , H. Zhang , et al. 2025. “Thresholds for the Relationships Between Soil Trace Elements and Ecosystem Multifunctionality in Degraded Alpine Meadows.” Agriculture Ecosystems & Environment 391: 11. 10.1016/j.agee.2025.109743.

[ece373238-bib-0081] Xue, Y. , Z. J. Wu , L. L. Zhang , P. Gong , X. X. Dong , and Y. X. Nie . 2012. “Inhibitory Effect of DMPP on Soil Nitrification as Affected by Soil Moisture Content, pH and Organic Matter.” Ying Yong Sheng Tai Xue Bao 23, no. 10: 2663–2669. 23359924

[ece373238-bib-0082] Yuan, J. Y. , M. Sadiq , N. Rahim , et al. 2022. “Tillage Strategy and Nitrogen Fertilization Methods Influences on Selected Soil Quality Indicators and Spring Wheat Yield Under Semi‐Arid Environmental Conditions of the Loess Plateau, China.” Applied Sciences‐Basel 12, no. 3: 20. 10.3390/app12031101.

[ece373238-bib-0083] Zhang, K. Q. , Z. Zhu , R. R. Shi , N. R. Shi , Q. Tian , and X. M. Lu . 2024. “Phenotypic Diversity and Seed Germination of *Elaeagnus angustifolia* L. in Relation to the Geographical Environment in Gansu Province, China.” Agronomy 14, no. 9: 26. 10.3390/agronomy14092165.

[ece373238-bib-0084] Zhang, L. Y. 2014. “Morphological Characteristics and Germination Traits of Seeds in the Endangered Plant Lilium Tsingtauense.” Jiangsu Agricultural Sciences 42, no. 07: 193–194. 10.15889/j.issn.1002-1302.2014.07.232.

[ece373238-bib-0085] Zhang, P. , T. Zhang , and N. L. Chen . 2009. “Vertical Distribution Patterns of Soil Organic Carbon and Total Nitrogen and Related Affecting Factors Along Northern Slope of Qilian Mountains.” Ying Yong Sheng Tai Xue Bao 20, no. 3: 518–524. 19637585

[ece373238-bib-0086] Zhang, R. , K. Luo , D. L. Chen , et al. 2020. “Comparison of Thermal and Hydrotime Requirements for Seed Germination of Seven *Stipa* Species From Cool and Warm Habitats.” Frontiers in Plant Science 11: 10. 10.3389/fpls.2020.560714. 33101329 PMC7554346

[ece373238-bib-0087] Zhao, W. D. , L. F. Wang , Y. Song , H. L. Jiang , and X. L. Guo . 2026. “Leaf‐Fruit Trait Decoupling Along Environmental Gradients in Tropical Cryptocaryeae (Lauraceae).” Plants (Basel) 15, no. 1: 14. 10.3390/plants15010126. PMC12787473 41515071

[ece373238-bib-0088] Zhou, R. , M. L. Fan , M. Zhao , X. Q. Jiang , and Q. H. Liu . 2022. “Overexpression of *LtKNOX1* From *Lilium tsingtauense* in *Nicotiana benthamiana* Affects the Development of Leaf Morphology.” Plant Signaling & Behavior 17, no. 1: 12. 10.1080/15592324.2022.2031783. PMC9176240 35139775

[ece373238-bib-0089] Zhu, C. Y. , J. S. Li , A. L. Li , et al. 2024. “Analysis of Temporal and Spatial Changes of the Laoshan Bay Shoreline and Discussion on Ecological Restoration.” Marine Geology Frontiers 40, no. 9: 63–72. 10.16028/j.1009-2722.2023.090.

