# Supplementary material for: Variations in Seed and Fruit Traits of the Rare and Endangered Chinese Plant Lilium tsingtauense Along Environmental Gradients
Source: Ecol Evol. 2026 Mar 9;16(3):e73238. doi: 10.1002/ece3.73238 (PMC12971391; doi:10.1002/ece3.73238)
Supplement: Supplementary file 1 — Appendix S1: ece373238‐sup‐0001‐AppendixS1.docx. [file ECE3-16-e73238-s001.docx]

**SUPPLEMENT:**

**SCHEDULE 1** The results of detrended correspondence analysis of environmental factors

| Statistic | Axis 1 | Axis 2 | Axis 3 | Axis 4 |
| --- | --- | --- | --- | --- |
| Eigenvalues | 0.0074 | 0.0027 | 0.0006 | 0.0003 |
| Explained variation(cumulative) | 58.15 | 78.91 | 83.68 | 86.07 |
| Gradient length | 0.36 | 0.22 | 0.14 | 0.13 |

**SCHEDULE 2** RDA ordination results of seed and fruit traits and environmental factors of *Lilium tsingtauense*

| Statistic | Axis 1 | Axis 2 | Axis 3 | Axis 4 |
| --- | --- | --- | --- | --- |
| Eigenvalues | 0.3825 | 0.0299 | 0.0249 | 0.0028 |
| Explained variation (cumulative) | 38.25 | 41.24 | 43.73 | 44.01 |
| Pseudo-canonical correlation | 0.7104 | 0.4751 | 0.5752 | 0.3033 |
| Explained fitted variation (cumulative) | 86.82 | 93.60 | 99.26 | 99.89 |

**SCHEDULE 3** Contribution rate of environmental factors to seed and fruit traits of *Lilium tsingtauense*

| Name | Explains % | Pseudo-F | P |
| --- | --- | --- | --- |
| Elevation | 26.5 | 12.6 | 0.002 |
| Longitude | 7.9 | 4.1 | 0.032 |
| Soil water content | 2.5 | 1.3 | 0.278 |
| Latitude | 2.3 | 1.2 | 0.25 |
| Soil total phosphorus | 1.7 | 0.9 | 0.35 |
| Light intensity | 1.5 | 0.8 | 0.426 |
| Soil pH | 0.8 | 0.4 | 0.598 |
| Soil electrical conductivity | 0.5 | 0.2 | 0.722 |
| Soil organic matter | 0.4 | 0.2 | 0.866 |

**SCHEDULE 4** Stepwise regression analysis of seed and fruit traits and geographical environment factors of *Lilium tsingtauense* seeds

| Index | Regression equations | Standardized regression coefficients | R^2^ | P |
| --- | --- | --- | --- | --- |
| Nf | Nf=-112095.223+929.285Long+0.071Ele | B_Long_=0.351，B_Ele_=0.343 | 0.357 | 0.001 |
| Fl | Fl=26.202+0.091SWC | B_SWC_=0.429 | 0.184 | 0.008 |
| Fw | Fw=28.341-0.005Ele-4.111EC | B_Ele_=-0.473，B_EC_=-0.332 | 0.233 | 0.011 |
| Fsi | Fsi=0.988+0.273EC+0.0002Ele | B_EC_=0.479，B_Ele_=0.376 | 0.255 | 0.007 |
| Nsu | Nsu=79.852-0.017Ele-0.002Lig | B_Ele_=-0.394，B_Lig_=-0.367 | 0.375 | <0.001 |
| Ns | Ns=107.872-0.016Ele-0.003Lig+21.569EC | B_Ele_=-0.299，B_Lig_=-0.348，B_EC_=0.338 | 0.446 | <0.001 |
| TGW | TGW=24.962-50.827TP | B_TP_=-0.448 | 0.201 | 0.005 |
| GP | GP=26.931+0.052Ele+27.551EC | B_Ele_=0.703，B_EC_=0.309 | 0.450 | <0.001 |
